# A dose-ranging study of the bronchodilator effects of abediterol (LAS100977), a long-acting β_2_-adrenergic agonist, in asthma; a Phase II, randomized study

**DOI:** 10.1186/1471-2466-14-176

**Published:** 2014-11-14

**Authors:** Dave Singh, Helena Pujol, Anna Ribera, Beatriz Seoane, Eric Massana, Carol Astbury, Sandrine Ruiz, Gonzalo de Miquel

**Affiliations:** University of Manchester, Medicines Evaluation Unit Ltd, The Langley Building, Southmoor Road, Wythenshawe, Manchester M23 9QZ UK; Almirall Research and Development Centre, Barcelona, Spain

**Keywords:** LABA, Chronic respiratory disease, Asthma, Dose-finding, Bronchodilation

## Abstract

**Background:**

Long-acting β_2_-adrenergic agonists (LABAs) are recommended in combination with inhaled corticosteroids (ICSs) for asthma management. Abediterol is a novel, selective, potent, once-daily LABA in development for treatment of asthma and chronic obstructive pulmonary disease. This study aimed to determine abediterol doses with similar peak bronchodilatory effect to salbutamol 400 μg, and duration of action compatible with once-daily dosing in patients with persistent, stable asthma.

**Methods:**

This was a Phase II, randomized, double-blind, double-dummy, crossover, placebo-controlled, dose-ranging study (ClinicalTrials.gov NCT01425801) in 62 patients with mild-to-moderate asthma who were also receiving an ICS. Patients received single doses of abediterol 0.313, 0.625, 1.25, or 2.5 μg, salbutamol 400 μg, or placebo in the morning. Spirometry was performed up to 36 h post-dose; safety and tolerability were assessed throughout the study. The primary endpoint was change from baseline in peak forced expiratory volume in 1 s (FEV_1_). Additional endpoints included trough FEV_1_, normalized area under the FEV_1_ curve (FEV_1_ AUC) up to 24 h post-dose, and peak and trough forced vital capacity (FVC).

**Results:**

Abediterol produced dose-dependent improvements in peak FEV_1_ from baseline compared with placebo, from 0.274 (95% CI 0.221, 0.327) to 0.405 L (95% CI 0.353, 0.458) for abediterol 0.313 to 2.5 μg, respectively (p < 0.0001 all doses). Abediterol 0.625, 1.25, and 2.5 μg had similar magnitude of peak FEV_1_ effect to salbutamol. Dose-dependent changes from baseline in trough FEV_1_ versus placebo were 0.219 (95% CI 0.136, 0.302) to 0.400 L (95% CI 0.317, 0.483) for abediterol 0.313 to 2.5 μg, respectively (p < 0.0001). All abediterol doses achieved significant improvements versus placebo in FEV_1_ AUC 0–6, 0–12, and 0–24 h, and peak and trough FVC (p < 0.05). Less than 10% of patients experienced treatment-related adverse events for each dose of abediterol; most were mild to moderate in intensity and the most common were headache and nasopharyngitis. There were no clinically relevant changes in heart rate.

**Conclusions:**

Abediterol 0.625–2.5 μg provided dose-dependent, clinically and statistically significant bronchodilation versus placebo in patients with asthma, with a peak effect similar to salbutamol and duration of action compatible with once-daily dosing. All doses of abediterol were well tolerated.

**Electronic supplementary material:**

The online version of this article (doi:10.1186/1471-2466-14-176) contains supplementary material, which is available to authorized users.

## Background

The use of β_2_-adrenergic agonist bronchodilators in combination with inhaled corticosteroids (ICSs) to manage the symptoms of asthma is recommended by Global Initiative for Asthma (GINA) guidelines [1]. Currently licensed bronchodilators include salbutamol, a short-acting β_2_-adrenergic agonist (SABA) administered as required [2], salmeterol and formoterol, two long-acting β_2_-adrenergic agonists (LABAs) administered twice daily in combination with an ICS [3, 4], and the recently licensed vilanterol, a LABA administered once daily in fixed-dose combination with the ICS fluticasone furoate [5]. There is ongoing interest to develop once-daily medications to further simplify treatment regimens and improve patient compliance [6].

Abediterol is a novel, once-daily LABA in clinical development as a fixed-dose combination with an ICS for the treatment of asthma and chronic obstructive pulmonary disease (COPD) [7]. Abediterol displays high affinity for β_2_-adrenoceptors, plus a higher functional selectivity for β_2_-adrenoceptors over β_1_-adrenoceptors than formoterol, indacaterol, vilanterol and olodaterol in cellular models [7–9]. The potency of abediterol is also higher than salmeterol and indacaterol in isolated human bronchi and, in addition to these two LABAs, vilanterol and olodaterol in animal models [7, 10]. Abediterol has a rapid onset and prolonged duration of action [7, 10].

A Phase II clinical trial in patients with asthma provided results that were consistent with the preclinical data; abediterol resulted in significant improvements in lung function at 5 min post-dose that were greater than improvements seen with salmeterol, and significant bronchodilation was sustained up to 24 h post-dose [11]. These characteristics make abediterol an ideal candidate for once-daily dosing. Moreover, abediterol has a favorable cardiovascular safety and tolerability profile: in anesthetized dogs, abediterol had a lesser effect on heart rate compared with salmeterol, formoterol, and indacaterol at concentrations that produced comparable bronchodilation [7, 12]. In early phase clinical trials, doses of abediterol ≤10 μg were safe and well tolerated and did not demonstrate any clinically relevant effects on heart rate in healthy subjects [13], patients with asthma [14], or patients with COPD [15].

Drug regulatory authorities have concerns that excessively high LABA doses may cause unacceptable side effects [16]. The trough forced expiratory volume in 1 s (FEV_1_), measured 24 h after dosing, is an important endpoint to assess once-daily LABA efficacy [16]. The maximum effect achieved soon after dosing, otherwise known as the ‘peak’ effect, is also of interest for efficacy and safety reasons. A very high peak effect may indicate high levels of acute drug exposure that could predispose patients to β-agonist-mediated side effects, such as cardiovascular adverse events, when receiving chronic treatment [17]. Moreover, the ideal pharmacologic characteristic of a once-daily LABA is a low ‘peak to trough ratio’, ensuring relatively stable lung function throughout the day [18].

The peak effect of salbutamol is well characterized, and causes acute bronchodilation without safety concerns. The bronchodilator response following single-dose administration of salbutamol (4 x 100 μg) is commonly used as one of the diagnostic tests for asthma, as recommended by a joint European Respiratory Society (ERS)/American Thoracic Society (ATS) taskforce [19]. This effect is, therefore, a reasonable benchmark for a novel LABA to achieve and this degree of acute bronchodilation is considered clinically meaningful. A major regulatory agency recommended that the peak effects of abediterol should be compared to those of salbutamol to guide dose selection for further clinical trials in order to avoid a dose that induces a much greater degree of bronchodilation than is necessary for clinical effectiveness, which could increase the potential for safety concerns during chronic treatment.

We report a placebo-controlled, dose-ranging, crossover study comparing the acute bronchodilator effects of abediterol to salbutamol in patients with persistent stable asthma. The primary aim of the study was to investigate the peak bronchodilatory effect of abediterol versus placebo and to confirm it was in the same range as that of salbutamol. The full profile of lung function over 24 h, including the trough FEV_1_, was also measured. The goal of this study was to enable the selection of abediterol doses for future long-term clinical trials, based on a peak effect similar to that of salbutamol 400 μg metered dose inhaler (MDI), coupled with a trough effect compatible with a 24-h duration of action. This trial design was a specific recommendation by a major regulatory agency.

## Methods

### Study design

This was a Phase IIa, randomized, double-blind, double-dummy, 6-way crossover, single-dose, multicenter, dose-ranging study conducted between August 2011 and January 2012. The study was conducted in two sites in the UK and seven in Germany. The study protocol was approved by an independent ethics committee in each country (see Additional file 1) and complied with the Declaration of Helsinki and the International Conference on Harmonisation and Good Clinical Practice guidelines. Patients provided written, informed consent.

Following a screening visit and a 12–16 day run-in period to assess clinical stability, participants were randomized to 1 of 6 treatment sequences according to a balanced Williams design for crossover studies (1:1:1:1:1:1). Block randomization was performed by the Sponsor, using a computer-generated schedule to assign a treatment sequence to each patient randomization number. The block size and randomization schedule were not communicated to investigators. Patients received abediterol 0.313, 0.625, 1.25, or 2.5 μg, salbutamol 400 μg (administered as four inhalations with 100 μg per inhalation), or placebo (at 09:00 h ±1 h) at each visit, with a washout period of 7–14 days. A follow-up telephone call to assess safety was made approximately 2 weeks following the final treatment visit or premature discontinuation.

Abediterol and placebo to abediterol were administered via a multidose dry powder inhaler (Genuair^®^). Salbutamol and placebo to salbutamol were administered via a pressurized MDI (Ventolin™ Evohaler™). In order to preserve study blinding, independent, trained personnel prepared the medication, delivered the device to patients, and collected the device following inhalation.

### Patients

Eligible participants were men or women aged 18–70 years with a clinical diagnosis of persistent asthma (according to GINA guidelines) for ≥6 months prior to screening. Eligible patients had an FEV_1_ >60% and ≤85% of the predicted normal value, with an FEV_1_ reversibility ≥12%, and an absolute increase in FEV_1_ of ≥200 mL over their baseline value following inhalation of salbutamol 400 μg at screening. Comparable FEV_1_ measurements were required at screening and pre-dose at each visit in order to ensure there were no carry-over effects from previous treatment periods (limit of variability ±20%). At enrollment, all patients were using only an ICS to control their asthma, and had been on a stable dose and regimen for at least 4 weeks prior to screening, up to the equivalent of 1600 μg/day of beclometasone dipropionate, and had not previously been exposed to abediterol.

Exclusion criteria included: a smoking history within the past 6 months or >10 pack-years; presence or history of relevant pulmonary disease or any other clinically relevant disease or abnormality as judged by the investigator; difficult-to-treat asthma; any respiratory tract infection ≤6 weeks prior to screening; hospitalization or emergency room treatment for asthma ≤3 months prior to screening; treatment with β_2_-antagonists; and positive laboratory tests for illicit drugs.

The use of anticholinergics, SABAs (other than salbutamol), LABAs, continuous oral or parenteral corticosteroids, methyl-xanthines, cromolyn sodium, nedocromil, leukotriene modifiers, anti-immunoglobulin E, β_1_-blocking agents, and other investigational drugs was prohibited during the study. Patients using prohibited medications underwent up to 6 weeks’ washout before the screening visit. Salbutamol pressurized MDI (100 μg/puff) was the only reliever medication permitted and was used on an as-needed basis and avoided 6 h prior to and during a treatment visit unless absolutely necessary.

### Efficacy assessments

Pulmonary function tests were conducted at screening, pre-dose, and at 0.25, 0.5, 1, 2, 3, 4, 6, 8, 12, 14, 23, 24, and 36 h post-dose at each treatment visit using standardized spirometers at each investigational site (eResearchTechnology, Philadelphia, PA, USA). Prior to the first spirometry measure, the technician demonstrated the procedure and allowed the patient two practice attempts. At each study time point, the maneuvers were repeated until three technically adequate measurements were made according to ERS/ATS guidelines for acceptability and repeatability [20], with a maximum of eight attempts allowed.

### Safety and tolerability assessments

Treatment-emergent adverse events (TEAEs), including serious adverse events, were recorded throughout the study and follow-up period. A physical examination was performed at screening and at 36 h post-dose following the last treatment period (Visit 6), and blood pressure and 12-lead electrocardiogram measures were made at screening, pre-dose, and at 0.75, 2, 6, 24 h post-dose for each treatment period, and 36 h post-dose at Visit 6. Clinical laboratory tests (blood chemistry, hematology, urinalysis, and a serum pregnancy test) were performed at screening and at 36 h post-dose at Visit 6. Blood glucose and serum potassium levels were assessed at screening, pre-dose, and at 4, 24 h post-dose for each treatment period, and 36 h post-dose at Visit 6.

### Statistics

The primary efficacy endpoint was change from baseline in peak FEV_1_ (defined as the highest FEV_1_ value observed within 4 h of the administration of the investigational medicinal product [IMP]) on Day 1. Secondary endpoints included change from baseline in peak forced vital capacity (FVC) on Day 1, trough FEV_1_ (defined as the highest average of the maneuvers performed at 23–24 h post IMP administration) and FVC on Day 2, change from baseline in FEV_1_ and FVC at each time point up to 36 h post-dose, and change from baseline in normalized FEV_1_ area under the curve (AUC; defined as the area between baseline FEV_1_ and the FEV_1_ curve, from 0 to × hours divided by × hours) at 0–6, 0–12, and 0–24 h post-dose. In accordance with the GINA guidelines, a change in FEV_1_ of 200 mL was considered clinically relevant [1].

A population of 48 patients was necessary to achieve 80% power to detect a 150 mL change from baseline in peak FEV_1_ for any dose of abediterol compared with placebo. Assuming a 15% withdrawal rate, the protocol required at least 54 patients to be randomized.

All reported efficacy assessments were analyzed in the intention-to-treat population, defined as all randomized patients who took at least one dose of the IMP (placebo, abediterol, or salbutamol) and had at least one baseline and one post-dose FEV_1_ value for at least one treatment visit. Safety analyses are reported in the safety population, which consisted of all randomized patients who took at least one dose of the IMP. All statistical analyses were performed using Statistical Analysis System (SAS Institute Inc., Cary, NC, USA) version 9.1.3 software.

Efficacy variables were analyzed using an analysis of covariance model for crossover designs, with fixed-effect factors of sequence, treatment, and period, patient within sequence as a random effect, and baseline FEV_1_ or FVC as a covariate, as appropriate. Between-treatments comparisons were performed by means of contrasts on the treatment factor. The difference between treatments was estimated by the difference between the least squares means. Statistical comparisons between active treatments and placebo were two-sided hypothesis tests with a significance level of 0.05.

## Results

### Study population

**Figure 1:**
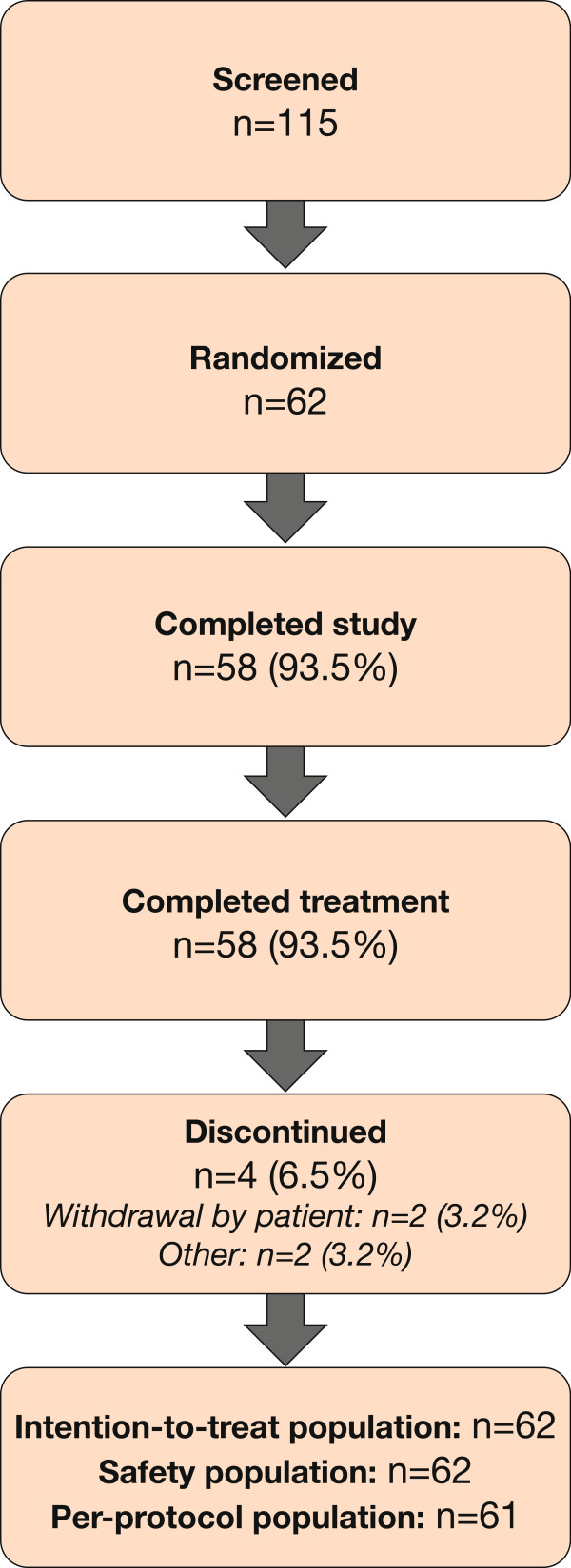
**Diagram of patient flow.**

A total of 115 patients were screened, with 62 patients randomized (40/62 [64.5%] male, mean age 40.9 ± 11.2 years). Patient demographics are presented in Table 1. A diagram of patient flow is presented in Figure 1. All 62 patients were included in the intention-to-treat and safety populations. One protocol violation occurred when a patient was inadvertently given the treatment scheduled for Visit 6 at Visit 5, and vice versa. Data for this patient were analyzed according to the treatments received. Four patients withdrew or were discontinued from the study: two due to inability to comply with the study schedule, one who did not fulfill stability criteria, and one for unknown reasons (patient did not return for the premature discontinuation visit).

**Table 1:** **Patient demographics at baseline: safety population**

	Patients (N = 62)
Age, mean no. years (SD)	40.9 (11.2)
Male gender, n (%)	40 (64.5)
Race, n (%)	
White	58 (93.5)
Other	4 (6.5)
Cigarette smoking status, n (%)	
Former smoker	21 (33.9)
Never smoked	41 (66.1)
Smoking history, mean no. pack-years (SD)	4.6 (3.3)
Asthma duration, mean no. years (SD)	25.5 (13.2)
Asthma severity, n (%)	
60% ≤ FEV_1_ < 80% of predicted	46 (74.2)
FEV_1_ ≥80% of predicted	16 (25.8)
Bronchial reversibility^a^, mean % (SD)	21.3 (8.2)
FEV_1_ absolute reversibility^b^, mean L (SD)	0.568 (0.261)

^a^calculated as 100 x (FEV_1_ [post-bronchodilator] – FEV_1_ [pre-bronchodilator]/FEV_1_ [pre-bronchodilator]); ^b^calculated as FEV_1_ (post-bronchodilator) – FEV_1_ (pre-bronchodilator).

FEV_1_, forced expiratory volume in 1 s; SD, standard deviation.

### Efficacy

#### ***Peak FEV***_***1***_

**Figure 2:**
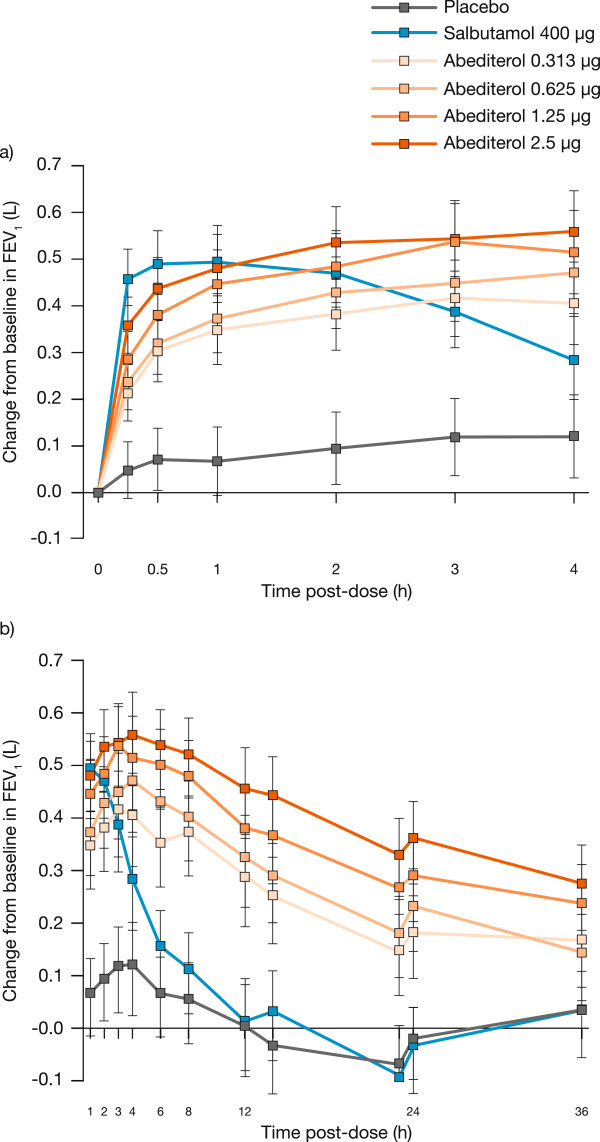
**Change from baseline in FEV**_**1**_ **over time.** Data are presented as least squares means with 95% confidence intervals: **a)** p < 0.0001 versus placebo for salbutamol and all doses of abediterol at all time points; **b)** p < 0.0001 for all doses of abediterol 0–24 h post-dose and abediterol 1.25 and 2.5 μg 36 h post-dose versus placebo, p = 0.002 and p = 0.01 for abediterol 0.313 and 0.625 μg, respectively, at 36 h post-dose versus placebo. FEV_1_, forced expiratory volume in 1 s.

**Figure 3:**
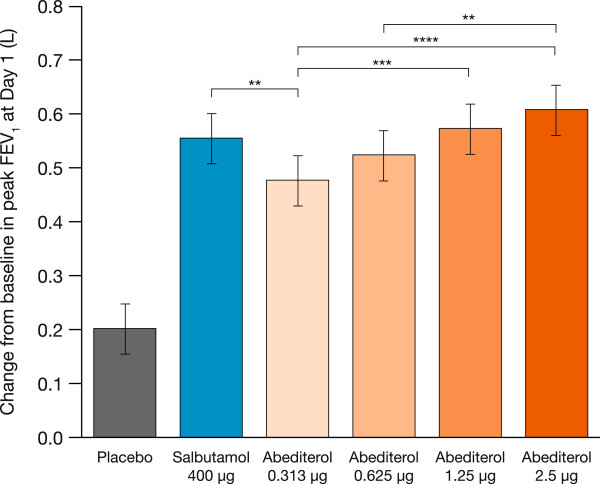
**Change from baseline in peak FEV**_**1**_ **at Day 1.** Data are presented as least squares means ± standard error: p < 0.0001 versus placebo for all doses of abediterol and salbutamol 400 μg; **p < 0.01, ***p < 0.001, ****p < 0.0001 (analysis of covariance). FEV_1_, forced expiratory volume in 1 s.

Mean baseline FEV_1_ values were similar across all treatment periods, ranging from 2.63 L to 2.69 L. The full profile of lung function after each treatment is shown in Figure 2. The onset of action of abediterol was rapid, with significant bronchodilation compared with placebo observed at the first study time point (15 min) post-dose (p < 0.0001 for all doses of abediterol versus placebo, Figure 2a). All doses of abediterol produced significantly greater improvements in peak FEV_1_ from baseline compared with placebo (p < 0.0001 for all doses, Table 2), and the magnitude of effect of abediterol 0.625, 1.25, and 2.5 μg was not significantly different from salbutamol 400 μg (Figure 3, Table 3). Least squares means differences in change from baseline in peak FEV_1_ versus placebo were 0.274, 0.322, 0.371, and 0.405 L for abediterol 0.313, 0.625, 1.25, and 2.5 μg, respectively, and 0.353 L for salbutamol 400 μg (Table 3). Median time to peak FEV_1_ was 3 h post-dose for abediterol (all doses), 2 h post-dose for placebo, and 1 h post-dose for salbutamol 400 μg.

**Table 2:** **Change from baseline in pulmonary function parameters (L): intention-to-treat population**

	Placebo N = 59	Salbutamol 400 μg N = 58	Abediterol 0.313 μg N = 60	Abediterol 0.625 μg N = 60	Abediterol 1.25 μg N = 60	Abediterol 2.5 μg N = 61
Peak FEV_1_	0.202 (0.045)	0.555**** (0.045)	0.477****^††^ (0.045)	0.524**** (0.045)	0.573**** (0.045)	0.608**** (0.045)
Trough FEV_1_^a^	-0.054 (0.039)	-0.076 (0.039)	0.166****^††††^ (0.039)	0.205****^††††^ (0.039)	0.278****^††††^ (0.039)	0.346****^††††^ (0.039)
Normalized FEV_1_ AUC_0–6_	0.088 (0.039)	0.347**** (0.039)	0.354**** (0.038)	0.403****^†^ (0.038)	0.461****^††††^ (0.038)	0.496****^††††^ (0.038)
Normalized FEV_1_ AUC_0–12_	0.063 (0.040)	0.219**** (0.040)	0.348****^††††^ (0.039)	0.393****^††††^ (0.039)	0.455****^††††^ (0.039)	0.496****^††††^ (0.040)
Normalized FEV_1_ AUC_0–24_	0.007 (0.039)	0.100** (0.039)	0.282****^††††^ (0.039)	0.321****^††††^ (0.038)	0.390****^††††^ (0.038)	0.446****^††††^ (0.039)
Peak FVC	0.222 (0.034)	0.361**** (0.034)	0.317** (0.034)	0.329*** (0.034)	0.361**** (0.034)	0.380**** (0.034)
Trough FVC^a^	-0.028 (0.035)	-0.065 (0.035)	0.094**^††††^ (0.035)	0.050*^††^ (0.035)	0.111***^††††^ (0.035)	0.120***^††††^ (0.035)

Data are least squares means (standard error).

^a^trough values are the mean of the 23 and 24 h post-dose measurements.

*p < 0.05, **p < 0.01, ***p < 0.001, ****p < 0.0001 versus placebo; ^†^p < 0.05, ^††^p < 0.01, ^††††^p < 0.0001 versus salbutamol 400 μg (analysis of covariance).

AUC, area under the FEV_1_ curve; FEV_1_, forced expiratory volume in 1 s; FVC, forced vital capacity.

**Table 3:** **Comparison of peak FEV**_**1**_ **for abediterol versus placebo and abediterol versus salbutamol**

	Least–squares mean difference (standard error)	95% confidence interval
Abediterol 2.5 μg vs. placebo	0.405 (0.027)	0.353, 0.458
Abediterol 1.25 μg vs. placebo	0.371 (0.027)	0.318, 0.424
Abediterol 0.625 μg vs. placebo	0.322 (0.027)	0.269, 0.375
Abediterol 0.313 μg vs. placebo	0.274 (0.027)	0.221, 0.327
Salbutamol 400 μg vs. placebo	0.353 (0.027)	0.299, 0.406
Abediterol 2.5 μg vs. salbutamol 400 μg	0.0529 (0.0270)	-0.0002, 0.1061
Abediterol 1.25 μg vs. salbutamol 400 μg	0.0181 (0.0269)	-0.0350, 0.0711
Abediterol 0.625 μg vs. salbutamol 400 μg	-0.0308 (0.0269)	-0.839, 0.0223
Abediterol 0.313 μg vs. salbutamol 400 μg	-0.0783 (0.0269)	-0.1313, -0.0253

Data included for comparisons of all doses of abediterol with placebo and with salbutamol.

#### ***Trough FEV***_***1***_

**Figure 4:**
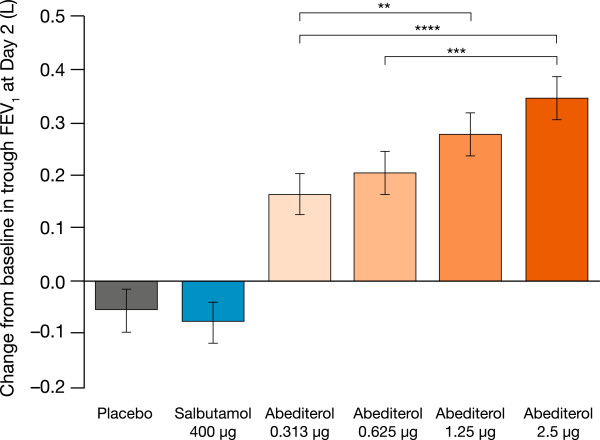
**Change from baseline in trough FEV**_**1**_ **at Day 2.** Data are presented as least squares means ± standard error: p < 0.0001 versus placebo for all doses of abediterol; **p < 0.01, ***p < 0.001, ****p < 0.0001 (analysis of covariance). FEV_1_, forced expiratory volume in 1 s.

Statistically significant increases in FEV_1_ were sustained at 24 h post-dose compared with placebo (p < 0.0001 for all doses of abediterol 0–24 h post-dose, Figure 2b). Abediterol significantly improved trough FEV_1_ from baseline compared with placebo (p < 0.0001 for all doses; Figure 4). Least squares means differences in change from baseline in trough FEV_1_ versus placebo were 0.219 L (95% CI 0.136, 0.302), 0.259 L (95% CI 0.176, 0.342), 0.332 L (95% CI 0.249, 0.415), and 0.400 L (95% CI 0.317, 0.483) for abediterol 0.313, 0.625, 1.25, and 2.5 μg, respectively (Table 2). The effects of salbutamol on FEV_1_ were not sustained after 6 h post-dose, as expected for a SABA (Figure 2b).

#### ***FEV***_***1***_***AUC***

Change from baseline in normalized FEV_1_ AUC_0–6_, FEV_1_ AUC_0–12_, and FEV_1_ AUC_0–24_ was significantly higher for all doses of abediterol versus placebo (p < 0.0001 for all, Table 2), with dose-related effects observed. The change from baseline in normalized FEV_1_ AUC_0–6_ was significantly greater for abediterol 0.625–2.5 μg versus salbutamol 400 μg (p < 0.05, Table 2).

#### FVC

Mean baseline FVC values were comparable across treatment periods, ranging from 4.23 L to 4.30 L. Abediterol produced significantly greater change from baseline in peak FVC compared with placebo (p < 0.01 for all doses, Table 2). The change from baseline in peak FVC was similar to that observed with salbutamol. Additionally, abediterol significantly improved trough FVC from baseline compared with placebo (p < 0.05 for all doses, Table 2). All doses of abediterol significantly (p < 0.05) increased FVC versus placebo up to 24 h post-dose except for the 0.625 μg dose at 12 and 23 h.

### Safety and tolerability

The percentage of patients experiencing TEAEs was similar across all treatment groups (Table 4). Most TEAEs were mild to moderate in intensity, no patient discontinued the study due to a TEAE, and there were no serious adverse events or deaths. The most common TEAEs were headache and nasopharyngitis, reported by 41.9 and 27.4% of patients, respectively. The percentage of patients that experienced at least one treatment-related TEAE was <10% for all doses of abediterol, and was similar to that of salbutamol and placebo. No clinically significant changes in serum glucose or potassium concentrations were observed over time in any of the treatments groups (Table 5). Minor variations in heart rate over time were observed for all treatment groups, including placebo; none were considered to be dose related or clinically relevant (Table 5). There were no clinically relevant changes in clinical laboratory tests, blood pressure, or electrocardiogram parameters.

**Table 4:** **TEAEs occurring in ≥2% of patients in any treatment group: safety population**

	Number (%) of patients reporting TEAEs						
	Placebo N = 59	Salbutamol 400 μg N = 58	Abediterol 0.313 μg N = 60	Abediterol 0.625 μg N = 60	Abediterol 1.25 μg N = 60	Abediterol 2.5 μg N = 61	Total N = 62
Any TEAE	16 (27.1)	12 (20.7)	18 (30.0)	16 (26.7)	18 (30.0)	23 (37.7)	51 (82.3)
Headache	7 (11.9)	6 (10.3)	4 (6.7)	7 (11.7)	8 (13.3)	7 (11.5)	26 (41.9)
Nasopharyngitis	1 (1.7)	0	8 (13.3)	3 (5.0)	4 (6.7)	2 (3.3)	17 (27.4)
Chest discomfort	2 (3.4)	0	1 (1.7)	0	0	2 (3.3)	5 (8.1)
Wheezing	1 (1.7)	2 (3.4)	1 (1.7)	0	1 (1.7)	0	4 (6.5)
Oropharyngeal pain	0	1 (1.7)	0	1 (1.7)	0	3 (4.9)	4 (6.5)
Cough	2 (3.4)	0	0	1 (1.7)	0	0	3 (4.8)

TEAE, treatment-emergent adverse event.

**Table 5:** **Change in clinical safety parameters from pre-dose values following treatment: safety population**

	Placebo N = 59	Salbutamol 400 μg N = 58	Abediterol 0.313 μg N = 60	Abediterol 0.625 μg N = 60	Abediterol 1.25 μg N = 60	Abediterol 2.5 μg N = 61
Serum glucose (mmol/L)						
4 hours post-dose	-0.37 (0.56)	-0.24 (0.55)	-0.19 (0.71)	-0.27 (0.70)	-0.16 (0.94)	-0.23 (0.74)
24 hours post-dose	0.01 (0.38)	0.00 (0.33)	0.13 (0.51)	0.06 (0.62)	0.11 (0.45)	0.15 (0.59)
Serum potassium (mmol/L)						
4 hours post-dose	-0.01 (0.36)	-0.09 (0.38)	-0.01 (0.35)	0.10 (0.37)	0.02 (0.36)	-0.04 (0.35)
24 hours post-dose	-0.02 (0.37)	0.04 (0.34)	0.05 (0.33)	0.15 (0.32)	0.04 (0.28)	0.05 (0.38)
Heart rate (bpm)						
0.75 h post-dose	-0.6 (9.1)	2.0 (10.1)	-0.7 (6.1)	-0.8 (7.5)	-1.5 (9.0)	-0.8 (6.2)
2 h post-dose	-3.4 (8.7)	-1.0 (8.0)	-2.0 (6.6)	-3.4 (8.1)	-4.3 (8.4)	-3.2 (6.7)
6 h post-dose	5.1 (10.2)	7.2 (7.7)	6.2 (7.1)	6.2 (8.0)	6.4 (9.8)	5.3 (7.2)
24 h post-dose	-2.1 (9.8)	-1.9 (8.0)	-1.6 (6.7)	-0.8 (8.4)	-0.9 (8.7)	-0.6 (7.6)

Data are mean (standard deviation).

bpm, beats per minute.

## Discussion

In this study, abediterol 0.313, 0.625, 1.25, and 2.5 μg provided clinically and statistically significant improvements in peak bronchodilation versus placebo in patients with persistent, stable asthma. Abediterol doses ≥0.625 μg also caused bronchodilation compatible with once a day administration, as improvements in trough FEV_1_ >250 mL compared with placebo were observed. The peak bronchodilatory effect of abediterol was similar to that of salbutamol 400 μg for all doses ≥0.625 μg.

A mean increase in peak FEV_1_ >200 mL is considered clinically relevant in asthma [1]. This magnitude of change was exceeded for all doses of abediterol, including the lowest dose, 0.313 μg. The primary objective of this study was to define abediterol doses that caused peak bronchodilation similar to salbutamol, the most widely prescribed SABA [21]. High doses of β_2_-agonists may increase the degree of bronchodilation but also can cause excessive systemic side effects through systemic absorption [22]. However, the current study demonstrates that the doses of abediterol from 0.625 to 2.5 μg induce acute bronchodilation similar to salbutamol, and therefore treatment with higher doses may not be warranted.

Significant bronchodilation compared with placebo was achieved with a range of abediterol doses from 0.313 to 2.5 μg, consistent with the high potency of abediterol demonstrated in preclinical studies that suggested a potential for significant bronchodilation *in vivo*[7]. Abediterol has higher potency than formoterol, salmeterol, indacaterol, olodaterol, and vilanterol in anesthetized guinea pigs [7, 10] as well as higher potency than salmeterol and indacaterol with comparable potency to formoterol in isolated human bronchi [7]. Based on the results of this preliminary dose-ranging study and the guidelines published by Chowdhury et al. [16], the clinically effective dose in asthma is considered most likely to be in the range of 0.625–2.5 μg.

Significant bronchodilation compared with placebo was observed for salbutamol and all doses of abediterol at the first-assessed time point (15 min) post-dose; this indicates a rapid onset of action for both salbutamol and abediterol. Salmeterol and formoterol, both twice-daily LABAs, are widely used as maintenance therapy in COPD and asthma due to their longer durations of action compared with salbutamol [6, 18]. In clinical studies in patients with asthma, the onset of action of formoterol 24 μg is consistently faster than that of salmeterol 50 μg, with a median onset of action of 3.6 min compared with 31.0 min in one study [23, 24]. *In vitro* experiments in isolated human bronchi suggest that the onset of action of abediterol is faster than salmeterol and is not significantly different from formoterol and indacaterol [7].

The prolonged duration of action of abediterol, with significant improvements in trough FEV_1_, normalized FEV_1_ AUC_0–24_, and trough FVC relative to placebo, and significant bronchodilation maintained up to 36 h post-dose is consistent with the results of previous clinical studies in healthy subjects and patients with COPD or asthma [11, 13, 25]. For example, a recent Phase II clinical study in patients with COPD demonstrated that abediterol 2.5, 5, and 10 μg achieved >200 mL increases in FEV_1_ at 24 h, and that this bronchodilatory effect was greater than that of indacaterol 150 μg, and the duration of action was similar [25]. Taken together, these results are compatible with a once-daily dosing regimen in asthma and COPD.

The convenience of once-daily dosing is likely to improve compliance in patients with asthma, leading to better patient outcomes [26, 27], as shown in various studies, including that of Wells et al. whereby once-daily ICS led to improved compliance in patients with asthma compared with ICS administered multiple times daily [28]. LABA monotherapy treatment is contra-indicated in asthma; co-administration with an ICS is mandated, preferably as a fixed-dose combination to reduce the risk of monotherapy administration. Development of once-daily LABAs for use in fixed-dose combinations with once-daily ICS is important for simplifying treatment regimens and, therefore, helping improve patient compliance and general asthma management.

The safety and tolerability profile of abediterol in this study is comparable to that reported in other studies in the abediterol development program [11–13, 25]. Single doses of abediterol 0.313–2.5 μg were safe and well tolerated, and the TEAEs were consistent with the known safety profile of β_2_-agonists.

## Conclusion

Abediterol 0.625–2.5 μg demonstrated a peak bronchodilatory response similar to that of a high dose of salbutamol (400 μg) at very low doses, and showed a clear dose–response relationship. Abediterol is a promising new, highly potent, LABA with a sustained duration of action and a favorable safety and tolerability profile. These results suggest that further studies are warranted to further evaluate the efficacy and safety of abediterol in combination with ICS in both asthma and COPD.

## Electronic supplementary material

Additional file 1: **Independent Ethics Committees (IEC).** (DOCX 17 KB)

## Abbreviations

ATS: American Thoracic Society
COPD: Chronic obstructive pulmonary disease
ERS: European Respiratory Society
FEV_1_ AUC: Area under the FEV_1_ curve
FEV_1_: Forced expiratory volume in 1 s
FVC: Forced vital capacity
GINA: Global Initiative for Asthma
ICS: Inhaled corticosteroid
IMP: Investigational medicinal product
LABA: Long-acting β_2_-adrenergic agonist
MDI: Metered dose inhaler
SABA: Short-acting β_2_-adrenergic agonist
TEAE: Treatment-emergent adverse event.
