# Supplementary material for: A dose-ranging study of the bronchodilator effects of abediterol (LAS100977), a long-acting β2-adrenergic agonist, in asthma; a Phase II, randomized study
Source: BMC Pulm Med. 2014 Nov 14;14:176. doi: 10.1186/1471-2466-14-176 (PMC4320624; doi:10.1186/1471-2466-14-176)
Supplement: Supplementary file 1 — Additional file 1: Independent Ethics Committees (IEC). (DOCX 17 KB) [file 12890_2014_647_MOESM1_ESM.docx]

**Additional File 1. Independent Ethics Committees (IEC)**

**Name and address of central IEC:**

- Germany:
  Ethikkommission der Landesärztekammer Hessen
  Im Vogelsgesang 3
  60488 Frankfurt am Main
- UK:
  North West – GM Central
  North West Centre of Research Ethics Committees
  Barlow House, 3^rd^ Floor
  4 Minushall St
  Manchester M1 3DZ

**Table 1: Principal investigator institution and name, address and chairperson of local IEC**

| **Country** | **Principal Investigator Institution** | **Name and address of local IEC** |
| --- | --- | --- |
| Germany | INSAF GmbH (Institut für Atemwegsforschung)  Biebricher Allee 34  65187 Wiesbaden | Ethikkommission der Landesärztekammer Hessen  Im Vogelsgesang 3  60488 Frankfurt am Main |
| Germany | MEDARS GMBH  Kaiserdamm 9  14057 Berlin | Ethikkommission des Landes  Berlin,  Landesamt für Gesundheit und  Soziales  Fehrbelliner Platz 1  10707 Berlin |
| Germany | Klin. Forschung Gruppe Nord GmbH (Klin. Forschung)  Georgenstraße 24  10117 Berlin | Ethikkommission des Landes  Berlin,  Landesamt für Gesundheit und  Soziales  Fehrbelliner Platz 1  10707 Berlin |
| Germany | PAREXEL International GmbH (Early Phase Clinical Unit)  Spandauer Damm 130 Haus 31  14050 Berlin | Ethikkommission des Landes  Berlin,  Landesamt für Gesundheit und  Soziales  Fehrbelliner Platz 1  10707 Berlin |
| Germany | Pneumologisches Forschungsinstitut am KH Großhansdorf  Wöhrendamm 80  22927 Großhansdorf | Ethikkommission der Ärztekammer Schleswig-Holstein  Bismarckallee 8-12  23795 Bad Segeberg |
| Germany | Klinische Forschung Hamburg GmbH  Hoheluftchaussee 18  20253 Hamburg | Ethikkommission der  Ärztekammer Hamburg  Humboldtstr. 67a  22083 Hamburg |
| Germany | IKF Pneumologie  Stresemannallee 3  Schaumainkai 101-103  60596 Frankfurt | Ethikkommission der  Landesärztekammer Hessen  Im Vogelsgesang 3  60488 Frankfurt am Main |
| UK | Wythenshawe University Hospital of South Manchester  Southmoor Rd  Manchester  M23 9LT | Northwest 6 REC  Greater Manchester South  Barlow House, 3^rd^ Floor  4 Minshull St  Manchester  M1 3DZ |
| UK | Queen Anne Street Medical Centre (Nephrology)  18-22 Queen Anne Street  London  W1G 8HU | London - Fulham REC  Charing Cross Hospital  Fulham Palace Road  London  W6 8RF |

IEC, Independent Ethics Committee
